# Addition of Bases to the 5'-end of Human Telomeric DNA: Influences on Thermal Stability and Energetics of Unfolding

**DOI:** 10.3390/molecules19022286

**Published:** 2014-02-21

**Authors:** Katherine L. Hayden, David E. Graves

**Affiliations:** 1 Department of Chemistry, University of Alabama at Birmingham, Birmingham, AL 35294, USA; E-Mail: klanier@uab.edu; 2 Comprehensive Cancer Center, University of Alabama at Birmingham, Birmingham, AL 35294, USA

**Keywords:** telomere, G-quadruplex, stability, circular dichroism (CD), differential scanning calorimetry (DSC), pressure perturbation calorimetry (PPC), osmolyte perturbation

## Abstract

Telomeric DNA has been intensely investigated for its role in chromosome protection, aging, cell death, and disease. In humans the telomeric tandem repeat (TTAGGG)_n_ is found at the ends of chromosomes and provides a novel target for the development of new drugs in the treatment of age related diseases such as cancer. These telomeric sequences show slight sequence variations from species to species; however, each contains repeats of 3 to 4 guanines allowing the G-rich strands to fold into compact and stable nuclease resistant conformations referred to as G-quadruplexes. The focus of this manuscript is to examine the effects of 5'-nucleotides flanking the human telomeric core sequence 5'-AGGG(TTAGGG) _3_-3' (h-Tel22). Our studies reveal that the addition of the 5'-flanking nucleotides (5'-T, and 5'-TT) results in significant changes to the thermodynamic stability of the G-quadruplex structure. Our data indicate that the observed changes in stability are associated with changes in the number of bound waters resulting from the addition of 5'-flanking nucleotides to the h-Tel22 sequence as well as possible intermolecular interactions of the 5' overhang with the core structure.

## 1. Introduction

Recent studies by Chaires and coworkers have demonstrated that the addition of bases to the 3' and 5'-end of the human telomeric (hTel) sequence (5'-AGGG(TTAGGG)_3_-3') results in significant changes to the thermal stability of the G-quadruplex [1]. To discern the thermodynamic effects resulting from the influence of 5'- overhangs on the core telomere stability, our laboratory utilized a variety of biophysical approaches including molecular dynamics simulations (MD), circular dichroism spectroscopy (CD), differential scanning calorimetry (DSC), osmotic stress perturbation, and pressure perturbation calorimetry (PPC). Preliminary characterization of the sequences shown in Table 1 were performed by DSC in the presence of K^+^ cations to determine influences in enthalpy and free energy of G-quadruplex unfolding resulting from the addition of the 5'-nucleotides. As demonstrated from both X-ray diffraction [2] and NMR [3,4], G-quadruplex structures are highly dependent on the type of cation present in the environment (either Na^+^ or K^+^) [5,6,7,8]. The studies described here are performed in a K^+^ environment since it is the higher concentration cation species within the cell and therefore the more intracellular biologically relevant structure. To ensure that each of the sequences were folded in the proper orientation (mixed parallel G-quadruplex) as predicted for the human telomeric sequence in the presence of K^+^ cation preliminary structural analyses for each sequence was performed using circular dichroism spectropolarimetry (CD) [9,10,11]. 

**Table 1:** Summary of thermal denaturation data derived from DSC experiments.

Name	Sequence	^a^ ΔH_tot_ (kJ/mol)	^b^ ΔΔH_tot_	^c^ T_m(tot)_ (°C)	^d^ ΔH_(1)_ (kJ/mol)	^e^ T_m(1)_ (°C)	^d^ ΔH_(2)_ (kJ/mol)	^e^ T_m(2)_ (°C)
22mer	5'-AGGG(TTAGGG)_3_-3'	154.5	NA	67.2	57.4	53.5	98.9	67.2
23mer	5'-TAGGG(TTAGGG)_3_-3'	189.2	34.7	62.4	72.3	52.3	98.5	66.5
24mer	5'-TTAGGG(TTAGGG)_3_-3'	229.7	75.2	61.3	60.0	46.2	174.3	62.4

^a^ The overall change in enthalpy of unfolding (ΔH_tot_); ^b^ the relative difference in the overall change in enthalpy of unfolding as compared to 22mer (ΔΔH_tot_); ^c^ the average melting temperature (T_m(tot)_) were determined by integrating the total area under the curve of the resultant thermograms; ^d^ The change in enthalpy of unfolding for each peak (ΔH_(1)_ and ΔH_(2)_); ^e^ the melting temperature for each peak (T_m(1)_ and T_m(2)_) were determined by fitting to a non-2-state model.

To further probe energetic changes that result from the addition of nucleotides to the 5' end of the h-Tel22 sequence, analyses to determine the influence of these dangling bases on the capture and/or release of water molecules upon unfolding of the G-quadruplex were performed. The interactions of solute molecules, such as water and counter ions, with each of the G-quadruplexes are influenced by alterations in loop geometries that result from the addition of the 5' nucleotides as well as intermolecular interactions of these nucleotides with the terminal G-tetrad. Ultimately, these additions may lead to changes in unfolding energetics resulting from the displacement of water (entropically favorable) and/or the breaking of hydrogen bonds (enthalpically unfavorable). To determine the change in the number of bound waters upon unfolding of the G-quadruplexes, DSC experiments were performed under conditions of increasing osmolyte concentrations [12,13,14,15,16]. This osmotic stress methodology provides considerable insight to the differences in solvation of the G-quadruplex resulting from the addition of nucleotides to the 5'-end of the h-Tel22 G-quadruplex. An alternative method of pressure perturbation calorimetry (PPC) was also utilized to determine changes in the partial specific volume for each of the three G-quadruplex DNAs (h-Tel22, 23-mer and 24-mer) resulting from the addition of 5'-T or 5'-TT to the 5'-AGGG(TTAGGG)_3_-3' human telomeric core sequence [17,18]. This method provided an accurate assessment of the change in the thermal expansion coefficient (α) upon thermal unfolding that can be used to calculate the changes in the partial specific volume of each sequence and correlate with changes in solvation. 

## 2. Results and Discussion

### 2.1. CD Analysis of G-Quadruplex Structures

**Figure 1:**
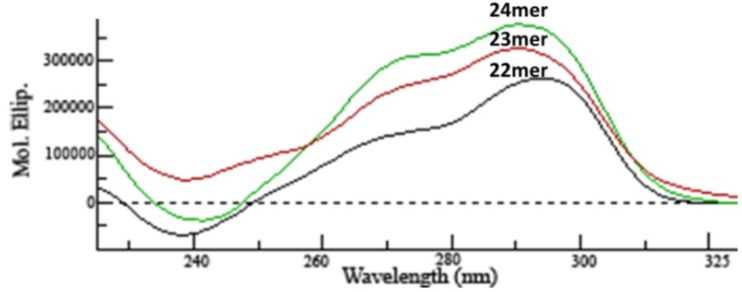
CD analysis was performed on a JASCO J-815 spectrometer at 5 μM strand concentrations in BPE-P. The parent h-Tel22 sequence (black) is 5'-AGGG(TTAGGG)_3_-3'. Addition of 5'-T forms the 23-mer (red) and addition of 5'-TT forms the 24-mer (green). Spectra were obtained at 25 °C.

The CD spectra for the parent 5'-AGGG(TTAGGG)_3_-3' human telomeric core sequence (h-Tel22) and both of the sequences containing 5'-overhangs (5'-TAGGG(TTAGGG)_3_-3' (23-mer) and 5'-TTAGGG(TTAGGG)_3_-3') (24-mer) are shown in Figure 1. The positive peaks at both 270 and 290 nm for each spectrum are indicative of the predicted mixed parallel quadruplex structure for the human telomere in potassium at 25 °C [4,17]. Examination of the CD spectra of the three G-quadruplex forming sequences (h-Tel22) and the 5'-T and 5'-TT (23-mer and 24-mer, respectively) reveal that the additional bases added to the 5-end of the 5'-AGGG(TTAGGG)_3_-3' does not disrupt or alter the mixed parallel G-quadruplex structures.

### 2.2. DSC Analysis of G-Quadruplex Stability

**Figure 2:**
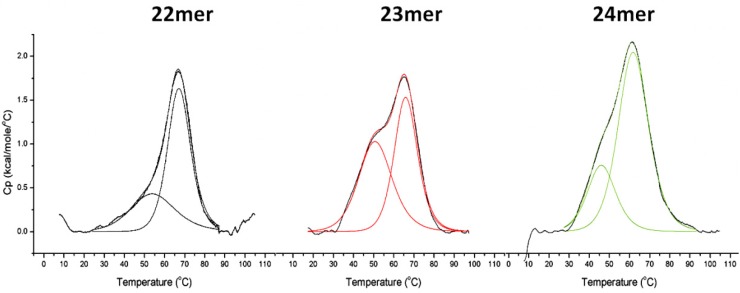
DSC unfolding of G-quadruplexes containing 5' base overhangs. DNA samples were prepared at 100 μM strand concentration in 10 mM potassium phosphate (pH 7.0), 1 mM EDTA and 100 mM KCl [BPE-P] buffer and analyzed using Microcal VP-DSC. Data were analyzed and processed using Origin DSC software provided by Microcal (GE Healthcare) and fitted using a non-2 state model. Examples of baseline adjusted raw data (black) and the non 2 state fit (color) are shown.

Upon addition of a 5'-T or 5'-TT to the h-Tel22 (22-mer) sequence resulting in the 23-mer and 24-mer, respectively, significant changes in the DSC thermograms were observed as shown in Figure 2. Data obtained from these DSC studies are provided in Table 1. The addition of dT and dTT results in a decrease in the average unfolding temperature (T_m(tot)_) of 5 to 6 °C as compared to the unfolding temperature of the parent h-Tel22 (22-mer) (67.2 °C for h-Tel to 62.4 and 61.3 °C for the 23-mer and 24-mer respectively).

The results for each of the thermograms reveal the influence of the addition of the 5'-T and 5'-TT nucleotides on the thermal unfolding profiles of the G-quadruplex structures. The unfolding thermogram for parent 22-mer G-quadruplex reveals two distinct unfolding domains, one at 53.5 °C and the other at 67.2 °C. When a 5'-T is added to the 5'-end of this sequence forming the 23-mer, we observe an apparent destabilization of the overall G-quadruplex as indicated by a decrease in the unfolding temperature of approximately 5 °C. When 5'-TT is added to the 5'-end of the 22-mer sequence forming the 24-mer, the unfolding temperature is decreased by an additional 1 °C to 61.3 °C. These data are indicative that the addition of 5'-T and 5'-TT dangling nucleotides to the 5'-end of the parent 22-mer results in significant energetic perturbations to the G-quadruplex structure.

In contrast, the enthalpy of unfolding (ΔH_tot_) as determined from the DSC experiments reveals significant increases upon addition of 5'-T and 5'-TT dangling nucleotides to the 5'-end of the parent 22-mer. Using DSC, the change in unfolding enthalpy for the parent 22-mer was found to be 154.5 kJ/mol. With the 5'-T dangling nucleotide, the change in unfolding enthalpy for the 23-mer was determined to be 189.2 kJ/mol. The addition of two thymines (5'-TT) to the 5'-end resulting in the 24-mer resulted in a change in unfolding enthalpy to 229.7 kJ/mol. This increase in the enthalpy of unfolding would indicate an apparent stabilization of the G-quadruplex structure despite the decreasing trend in the overall melting temperatures as the 5'-flanking nucleotides are added, contradicting the observed decrease in T_m_. In order to further explore this phenomenon and investigate the possible role of solvation *versus* intermolecular interactactions we turned to molecular dynamic simulations.

### 2.3. Molecular Dynamics Simulation on the Parent h-Tel22 and the 23-mer 24-mer G-quadruplexes

**Figure 3:**
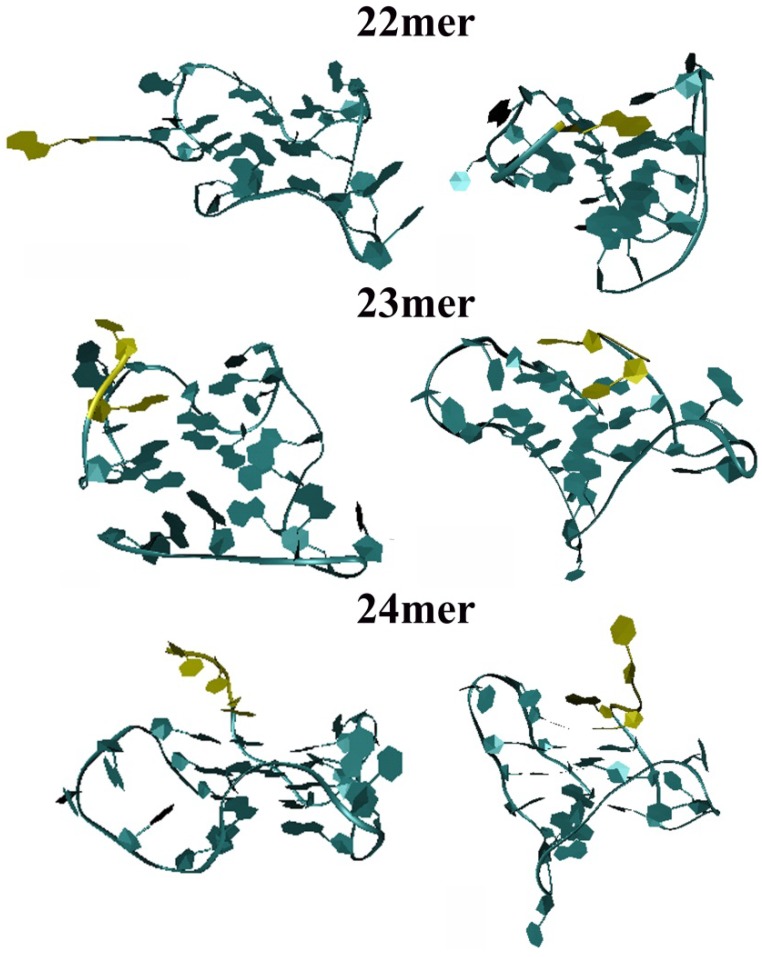
Images from molecular dynamic (MD) simulations (100 ps) of the parent 22-mer, the 5'-T (23-mer) and 5'-TT (24-mer) overhangs sequences based on Neidle’s crystal structure (left) and Patel’s solution structure (right). The positions of the 5'-A, 5'-TA and 5'-TTA overhang bases are shown in yellow while the remaining molecule is shown in blue.

To probe the conformational space available to the 5'-dangling nucleotides, molecular dynamic simulations were performed using two different starting structures. Both starting structures used in these simulations are G-quadruplex structures derived in potassium environments: the 22-mer (5'-AGGG(TTAGGG)_3_-3' G-quadruplex structure reported by Neidle and coworkers (PDB: 1KF1) and the NMR solution structure (5'-TAGGG(TTAGGG)_3_-3' reported by Patel and coworkers (PDB: 2GKU) [2,3]. Because both structures are remarkably different as can be seen in Figure 3, it was necessary to perform parallel MD calculations using both as starting structures. The observations obtained from the molecular dynamic simulations allowed us to gain insights into the observed changes in unfolding energies observed from the DSC studies and summarized in Table 1.

As revealed in Figure 3, the addition of nucleotides to the 5'-end of the h-Tel22 results in changes to the overall structure and stacking with the terminal G-tetrad. The 5'-flanking bases 5'-T for the 23-mer, and 5'-TT for the 24-mer may interact with G-tetrad guanines through the formation of additional hydrogen bonding, base stacking, and van der Waals interactions as seen in the NMR solution structure simulations (structures on the right) and X-ray crystal structure simulations (structures on the left) for the 22-mer, 23-mer and 24-mer. Data obtained from the molecular dynamic simulations corroborate the observed decreases in T_m_ found from the DSC studies due to possible weakening of the hydrogen bonding network as a result of interactions of the 5'-dangling ends with the G-tetrads. An example of this is seen for both the 23-mer and 24-mer solution and crystal structure simulations (Figure 3) where the 5'-adenine is oriented such that it can base stack and possibly even hydrogen bond with nearby guanines of the terminal G-tetrad of each quadruplex. Although this orientation could result in localized favorable interactions, the resulting interference of the G-tetrad may have a destabilizing effect on the overall structure since overall number of hydrogen bonds within the core tetrad structure would be reduced or distorted due to perturbation of the planar G-tetrad structure upon adenine base stacking. Since both the 23-mer and 24-mer 5'-overhangs would have similar adenine to G-tetrad interactions we would expect to similar decreases in T_m_ as compared to the 22-mer, which is what we observe in the DSC results of Table 1. At the same time, an increasing change in unfolding enthalpy is observed possibly resulting from a decrease in water interactions of the overall molecule to due to additional interactions of the 5' overhang with the neighboring TTA single-strand loop. The interactions of the 5'- overhang and the nearby TTA loops would release water molecules from the single strand overhangs, the interacting TTA loop, and the terminal G-tetrad between the loops. This release of these bound waters from the G-quadruplex is entropically favorable. These interactions are supported by MD simulations showing the additional 5'-thymines are favorably orientated to interact with a nearby TTA loop. The favorable interactions between the single strand loop and the 5'-dangling end may be driven by the displacement of localized water molecules upon interaction. Previous studies by Miyoshi and Olsen have demonstrated that as the solvation sphere about a G-quadruplex decreases the thermodynamic stability increases [18,19]. Similarly, Trent and coworkers report that increased hydration results in decreased stability of the G-quadruplex formed by the human telomeric sequence [20]. To further investigate the role of solvation in these quadruplexes, additional studies to be performed to measure changes in water uptake or release upon unfolding using osmotic perturbation and pressure perturbation calorimetry.

### 2.4. DSC with Osmotic Perturbation

The changes in T_m_ and unfolding enthalpies that are observed with the additions of the 5'-T and 5'-TT to the h-Tel22 G-quadruplex required a more extensive investigation of the change in number of water molecules associated with these sequences. As indicated by the molecular dynamic simulations, the interactions of water molecules with each of the quadruplex structures are predicted to be different due to changes in loop geometries as well as the accessibility of water molecules to the dangling 5'-ends of the G-quadruplexes. Each of the DNA sequences were examined by DSC under conditions of increasing osmolyte (glycerol) concentrations to determine the number of waters released and/or adsorbed upon unfolding [16,18,19,20,21]. A linear plot of the change in the apparent binding constant of water (K_a_) as calculated from the resultant DSC data *versus* the change in the concentration of water allows the determination of the change in the number of waters bound to the molecule upon melting from the slope of the straight line:

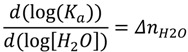
(1)

This study provides a quantitative approach to discern differences in solvation effects of the molecule as the 5'-T and 5'-TT overhangs were added to the core h-Tel22 sequence.

**Figure 4:**
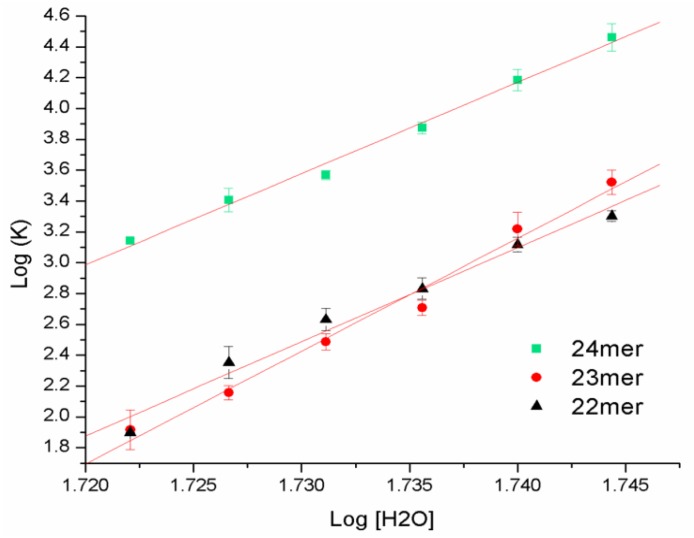
A plot of the change in the binding affinity of water (K_a_) *versus* the change in the concentration of water due to the increasing concentration of glycerol. From the slope of the line, one can estimate the number of waters absorbed or released upon unfolding. The absolute values of the slope are provided in Table 2.

The positive slopes of the data shown in Figure 4 demonstrate that all three sequences gain water molecules upon thermal unfolding. The h-Tel22 G-quadruplex (22-mer) is shown to gain 61 waters upon unfolding. Addition of the dangling 5'-T (23-mer) results in a gain of 73 waters upon unfolding, 12 additional waters as compared with the 22-mer. Interestingly, if an additional thymine is added (5'-TT) forming the 24-mer, the number of waters that are gained upon thermal denaturation drops back to the same amount that was observed for the parent h-Tel22 G-quadruplex. This indicates that the 23-mer containing the dangling 5'-TA is folded back onto the terminal G-tetrad of the G-quadruplex resulting in the formation of additional hydrogen bonds and/or stacking within the G-quadruplex rather than interacting with water molecules outside within the hydration sphere. The results observed from these studies may be further supported by pressure perturbation calorimetry (PPC), an analysis designed to experimentally determine the changes in partial specific volumes of the G-quadruplexes as they unfold.

**Table 2:** The number of waters bound upon thermal denaturation of 5' sequences.

Name	Sequence	R^2^	Δn*_w_*	Error
22-mer	5'-AGGG(TTAGGG)_3_-3'	0.9803	61.1	±4.3
23-mer	5'-TAGGG(TTAGGG)_3_-3'	0.9855	73.2	±4.4
24-mer	5'-TTAGGG(TTAGGG)_3_-3'	0.9916	59.2	±2.7

### 2.5. Pressure Perturbation Calorimetry (PPC)

Pressure perturbation calorimetry provides a novel method for determining the thermal expansion coefficient of macromolecules such as proteins and nucleic acids. This approach is applied to the G-quadruplex in an effort to determine the change in the partial volume of the G-quadruplex as it unfolds [7]. Unlike the constant pressure conditions of DSC, PPC is performed by alternating between high and low pressures while raising the temperature [22,23,24,25,26]. By changing the pressure above the liquid, we are able to induce entropy and heat changes such that:

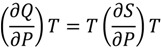
(2)

With a change in pressure and utilization of the Maxwell relationship we find that:

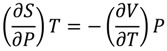
(3)

The following expression can then be derived and used to calculate the thermal expansion coefficient of the solute (α_s_) as:

*α_s_* = *α_o_* − *∆Q*/[*T∆Pm_s_V_s_*] (4)

**Figure 5:**
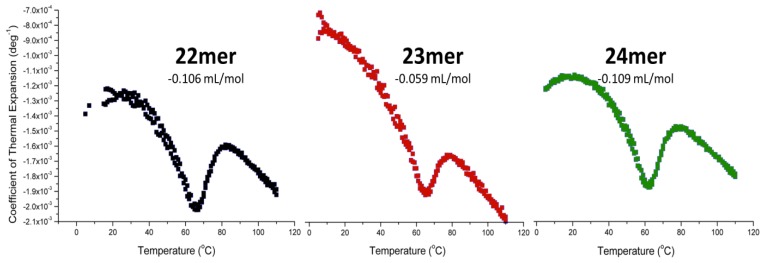
Pressure Perturbation Calorimetry (PPC) data for the thermal unfolding of the parent h-Tel22 (5'-AGGG(TTAGGG)_3_-3' (black), the 23-mer (5'-TAGGG(TTAGGG)_3_-3' (red) and the 24-mer (5'-TTAGGG(TTAGGG)_3_-3' (green) are analyzed using Origin PPC software provided by Microcal (GE Healthcare). Water/water, water/buffer and buffer/buffer scans are used to create a baseline for subtraction from the sample/buffer analysis. The data were integrated and normalized to concentration and molecular volume and baseline subtracted using the buffer/buffer scan. The area above the peak was integrated to yield the change in partial specific volume of the molecule upon thermal denaturation. The decrease in the change in specific volume of the parent h-Tel22 sequence (22-mer) was determined to be −0.106 mL/mol, the 5'-T (23-mer) was −0.059 mL/mol, and the (5'-TT) 24-mer was −0. 109 mL/mol.

As shown in Figure 5, a plot of the changes in the thermal expansion coefficient of the solute (α) *versus* the change in temperature results in a thermogram generating a peak similar to that of a normal DSC melt; however, integration of the area under (or above) allows one to quantitate of the change in the partial specific volume of the molecule upon unfolding. Because the volume for each deoxyoligonucleotide is constant, the resultant volume change represents the change in the volume of the solvation sphere for each specific sequence. As observed in Figure 5, each of the DNA sequences display negative changes in partial specific volume, indicating that the solvation sphere about the molecule is decreasing upon unfolding. Both the parent h-Tel22 sequence (22-mer) and the 24-mer (5'-TT) G-quadruplexes display similar decreases in volume, −0.106 mL/mol and −0.109 mL/mol, respectively. In contrast, the 23-mer (5'-T) sequence loses significantly less volume, −0.059 mL/mol than either the 22-mer or 24-mer G-quadruplexes. This observation is consistent with the increased water uptake by the 23-mer (13 additional water molecules) as compared with h-Tel22 and the 24-mer.

## 3. Experimental

Reagents used in all experiments were purchased from Fisher Scientific (Pittsburgh, PA, USA). HPLC purified nucleic acids were purchased from Midland Scientific (Midland, TX, USA) and used without further purification. Deoxy-oligonucleotides were solvated in 10 mM potassium phosphate, pH 7.0, 1 mM EDTA and 100 mM KCl (BPE-P) and strand concentrations verified for all experiments using UV-vis absorbance at 260 nm based on the extinction coefficients of 228,500 mol^−1^cm^−1^ (22-mer), 236,500 mol^−1^cm^−1^ (23-mer), and 244,600 mol^−1^cm^−1^ (24-mer).

### 3.1. Circular Dichroism Spectropolarimetry

Preliminary structural analysis for each G-quadruplex forming sequence was performed using circular dichroism (CD) spectropolarimetry to ensure that each sequence was indeed folded into the proper orientation (mixed-parallel quadruplex) as predicted for the human telomeric G-quadruplex in the presence of potassium cation. CD analysis for each of the DNA sequences (h-Tel22, 23-mer, and 24-mer) was performed on a J-815 spectrometer (JASCO Analytical Instruments, Easton, MD, USA) at 5 μM concentrations in BPE-P from 225 to 325 nm with a 4 s response delay at 25 °C. Each sequence was also analyzed in BPE-P buffer with 1 to 5% glycerol to ensure they were structurally robust throughout the DSC osmolyte perturbation analysis (data not shown).

### 3.2. Differential Scanning Calorimetry

The sequences provided in Table 1 were analyzed by DSC in the presence of K^+^ to determine the unfolding temperatures and the change in enthalpy and free energy of unfolding for each sequence. DSC analysis of each of the deoxyoligonucleotide sequences was performed on a Microcal VP-DSC (GE Healthcare Life Sciences, Pittsburgh, PA, USA) at 100 uM DNA in BPE-P. Each sample was analyzed from 5 to 110 °C at 60 °C/h. Results were cubic baseline adjusted and analyzed first by integration of the total area under the curve and second by fitting to a non-2-state model using Origin DSC analysis software provided by Microcal.

### 3.3. Molecular Dynamics Simulations

Molecular dynamics simulations were performed using AMBER (Version 12) [27]. Solvated simulations were performed on two different starting structures: The 22-mer sequence 5'-(AGGG(TTAGGG)_3_-3' structure derived from X-ray crystallography in the presence of K^+^ and the 23-mer (5'-TAGGG(TTAGGG)_3_-3') structure derived from high-resolution NMR in the presence of K^+^ [2,3]. Additional nucleotides were added to or removed from each starting structure using Discovery Studio, (Version 2.1, Accelrys, Inc., San Diego, CA, USA). Each structure was first minimized in vacuum before solvation using a 10 Å octagon TIP3PBOX. The solvated structures were then subjected to a series of explicit MD simulations to allow the surrounding waters to relax by slowly heating from 0 to 300 K while holding the deoxyoligonucleotide structures fixed with 5 kcal/mol*Å^2 ^harmonic constraint for 260 ps. The harmonic constraint was then dropped to 0.5 kcal/mol*Å^2^ for 60 ps while holding the volume constant. Finally, the harmonic constraint was completely removed for 100 ps during the final simulation.

### 3.4. Differential Scanning Calorimetry with Osmolyte Perturbation

Deoxyoligonucleotides (22-mer, 23-mer, and 24-mer) were dissolved and annealed in BPE-P at a stock concentration of 1 mM (strand). Samples of each strand were then dialyzed overnight in BPE-P with an appropriate glycerol percentage (ranging from 0 to 5% w/v), and diluted to 75 μM (strand) in appropriate buffer (containing the appropriate glycerol concentration) and degassed. Buffer and sample scans were heated from 5 to 105 °C at a rate of 1 °C/min. Each sample was analyzed a minimum of three times using freshly prepared DNA samples for each scan.

### 3.5. Pressure Pertrubration Calorimetry

Deoxyoligonucleotide solutions were prepared in BPE-P, degassed, and adjusted to 1 mM (strand) concentration. Samples were analyzed using a Microcal VP-DSC equipped with the pressure perturbation calorimetry unit (PPC) from 5 to 120 °C, where pulses of high pressure (70 psi) and low pressure (0 psi) were performed at every 1 °C with a 150 s delay between pulses. Scans of water against water, buffer against water, buffer against buffer, and sample against buffer were completed under identical conditions. Data were analyzed and fitted using Origin PPC software provided by Microcal.

## 4. Conclusions

The addition of nucleotides (5'-T, and 5'-TT) to the 5' end of the core human telomere sequence, 5'-AGGG(TTAGGG)_3_-3' (h-Tel22), results in significant changes to the thermodynamic, structural and hydration properties of these G-quadruplexes as compared with the h-Tel22 G-quadruplex. Overall, all three G-quadruplex structures (22-mer, 23-mer and 24-mer) maintain similar conformations as indicated by their respective CD spectra.

The thermal induced unfolding of the three deoxyoligonucleotides was examined by differential scanning calorimetry (DSC). The results of these experiments were found to be more complex than had been previously anticipated. The stabilizing effects of 5'-dangling nucleotides are well documented for duplex DNAs and RNAs [28,29]. In contrast, the incremental addition of dangling nucleotides (5'-T and 5'-TT) to the parent 5'-AGGG(TTAGGG)_3_-3' human telomeric core sequence (h-Tel22) results in a destabilization of 5 to 6 °C for the G-quadruplex structure. However, the enthalpy of unfolding (ΔH_tot_) was shown to increase in the presence of the 5'-dangling nucleotides. For the parent h-Tel22 (22-mer) G-quadruplex, the unfolding energy (ΔH_tot_) was determined to be 154.5 kJ/mol. Upon addition of the dangling 5'-T and 5'-TT, the resulting unfolding enthalpies were found to increase to 189.2 kJ/mol and 229.7 kJ/mol, respectively. The increase in unfolding enthalpy with addition of 5'-T and 5'-TT is thought to result from increasing intermolecular interactions between the 5'-dangling nucleotide(s) and a nearby TAA loop, as well as the additional water interactions upon unfolding of the G-quadruplex.

DSC studies were carried out under conditions of increasing osmolyte concentrations to probe the change in bound waters for the parent 22-mer, and the 23- and 24-mers. As shown in both Figure 4 and Table 2, the parent 22-mer G-quadruplex binds approximately 60 water molecules upon thermal denaturation. Upon the addition of the 5'-T, the 23-mer was found to bind 13 additional waters than that of the parent 22-mer G-quadruplex (72 waters total). Addition of two dangling 5'-nucleotides (5'-TT, forming the 24-mer) results in the change in the number of water molecules dropping back to 60, same as the parent 22-mer G-quadruplex. These data are in agreement with Trent and coworkers [22] who report hydration to be a major determinant of G-quadruplex stability and that increased hydration results in decreased stability. Although the osmolyte perturbation studies show that both the 22-mer and 24-mer sequences bind the same number of waters upon unfolding, these data do not indicate how many water molecules each sequence initially has bound in its native state prior to unfolding. If we consider that the decrease observed for the melting temperature of 24-mer relative to the parent 22-mer sequence is related to a destabilization brought on by an increase in solvent-accessible surface area, we may postulate that the 24-mer may have more water molecules associated in total, but that upon unfolding, the change in the number of waters to be the same amount as that observed for the parent 22-mer sequence. 

Using DSC-pressure perturbation calorimetry (PPC), the changes in the partial specific volumes for the parent 22-mer and the 23- and 24-mer sequences with 5'-dangling nucleotides were obtained. A negative peak upon unfolding indicates a decrease in the molecules’ partial specific volume. The PPC data relating changes in specific volumes are consistent with our observations for changes in water uptake. Both the 22-mer and the 24-mer exhibit comparable changes in specific volume of −0.106 mL/mol and −0.109 mL/mol, respectively, and have similar changes in water uptake (~60 water molecules). In contrast, the PPC data demonstrate that the 23-mer has both the smallest decrease in specific volume (−0.05 mL/mol) which is consistent with the largest uptake of water (72 water molecules); 13 more than either the parent 22-mer or the 24-mer with two 5'-TT dangling nucleotides. The 23-mer is found to bind more water molecules upon unfolding while at the same time having the smallest loss in partial specific volume, providing key insights into the nature of the 5'-dangling nucleotides with respect to solvent accessibility, interactions with a nearby TAA loop and/or base stacking with the terminal G-tetrad. 

The data provided by the molecular dynamics simulations on each of the sequences provide further insights into the conformational space that could be occupied by the 5'-dangling nucleotides as well as potential interactions of these dangling ends with the terminal G-tetrad and loop regions of the G-quadruplex. As previously described in Figure 3, the structural predictions from MD simulations indicate that the terminal 5'-A of the 22-mer sequence resides the majority of time away from the terminal G-tetrad and oriented toward the solvent. However, with the addition of a 5'-T, resulting the 23-mer, MD simulations predict that the 5'-T dangling nucleotide to be most stable when it is oriented into the G-quadruplex, resulting in the ability to form addition interactions with the G-tetrad (5'-A stack) and interactions with the TTA loop (H-bonding). Upon the addition of 5'-TT (creating the 24-mer sequence) MD simulations predicts that prior to unfolding, the 5'-dangling end to be oriented outside (similar to the 5'-A of the 22-mer) so that the 5'-TT has maximal interactions with water molecules in the solvation sphere. The results presented here demonstrate that subtle differences in 5'- ends of the G- quadruplexes plays significant and complex roles in modulating the overall structure and stability of this DNA structural motif. 
